# Influence of the child’s perceived general health on the primary caregiver’s health status

**DOI:** 10.1186/s12955-018-0840-z

**Published:** 2018-01-10

**Authors:** Janine Verstraete, Lebogang Ramma, Jennifer Jelsma

**Affiliations:** 1 Department of Physiotherapy, Red Cross War Memorial Children’s Hospital, Klipfontein Road, Rondebosch, Cape Town, 7700 South Africa; 2 Faculty of Health and Rehabilitation Sciences, Anzio Road, Observatory, Cape Town, 7925 South Africa

**Keywords:** HRQoL, Health related quality of life, Infants, Children, Caregivers, Cost utility analysis, EQ-5D

## Abstract

**Background:**

In estimating the impact of an intervention, ignoring the effect of improving the health of one member of the caregiver/child dyad on the Health Related Quality of Life (HRQoL) of the other member may lead to an underestimation of the utility gained. This may be particularly true for infants/young children and their caregivers. The aim of this study was to quantify the interaction between the child’s perceived general health as assessed by the newly developed Toddler and Infant Questionnaire (TANDI) on the reporting of the caregiver’s own HRQoL as assessed by the EQ-5D-3 L.

**Methods:**

A sample of 187 caregivers participated. A total of 60 caregivers of acutely-ill (AI) and 60 caregivers of chronically-ill (CI) children were recruited from a children’s hospital. The 67 caregivers of general population (GP) children were recruited at a pre-school. Each caregiver completed the proxy rating of their child’s HRQoL on the TANDI (The TANDI is an experimental HRQoL instrument, modelled on the EQ-5D-Y proxy, for children aged 1-36 months), which comprises of six dimensions of health and a rating of general health on a Visual Analogue Scale (VAS). The caregiver completed the EQ-5D-3 L, a self-report measure of their own HRQoL. Forward stepwise regression models were developed with 1) the VAS score of the caregiver and 2) the VAS score of the child as dependent variables. The independent variables for the caregiver included dummy variables for the presence or absence of problems on the EQ-5D-3 L and the VAS score of the child. The independent variables for the child included dummy variables for each TANDI dimension and the VAS of the caregiver.

**Results:**

The TANDI results indicated that in five of the six dimensions AI children had more problems than the other two groups and the GP children were reported to have a significantly higher VAS than the other two groups. The child’s VAS was significantly correlated with the caregiver’s VAS in all groups, but most strongly in the AI group. The preference based scores (using the UK TTO tariff) were only correlated in the AI group. The inclusion of the child’s VAS increased the variance accounted for 11% of the VAS score of the caregiver. Anxiety and depression was the only dimension which accounted for more variance (18%). Similarly the perceived health state, VAS of the caregiver accounted for 14% of the variance in the child’s VAS, second only to problems with play (25%).

**Conclusion:**

There does indeed appear to be a strong relationship between the VAS scores of the children and their caregivers. The perceived general health of the child influences the caregivers reporting of their general health, more than their own report of experiencing pain or discomfort or problems with mobility. Thus, improving the HRQoL of the very young child may improve the caregiver’s HRQoL as well. Conversely, if the caregiver has a lower perceived HRQoL this may result in a decrement in the reported VAS of the child, independent of the presence or absence of problems in the different dimensions. This improvement is not currently captured by Cost Utility Analysis (CUA). It is recommended that future research investigates this effect with regards to CUA calculations.

## Background

Cost Utility Analysis (CUA) can be calculated from a health care or societal perspective. CUA from a health care perspective is calculated as the ratio between the cost of a health programme or intervention and the benefit of it in term of the number of years the patient lives in full health [1–5]. CUA is often calculated to determine the effect that therapeutic intervention has on health or Health-Related Quality of Life (HRQoL). The burden of the health state is measured by the change in Quality Adjusted Life Years (QALYs) which takes into account quality, in terms of HRQoL utility values, and the quantity, or time spent, in a specific health condition. QALYs are measured on a scale between 0 (death) -1 (full health) where the intervals on the scale are equal and losses or gains on the scale can be aggregated [3, 4]. CUA can also adopt a societal perspective in which all societal costs and effects of health care management are included in the calculation, regardless of who experiences them [5, 6]. Ignoring the costs and consequences generated from a societal perspective, such as the provision of informal care, leads to health care decisions based on economic evaluations which have not considered all of the relevant information and could lead to underestimation of the utility gained [5–7].

There have been efforts to understand CUA from a societal perspective as well as to develop algorithms for these calculations. Bobinac et al. (2011), found that a change in the HRQoL of an individual may affect the HRQoL of significant others through the care-giving effect or the family effect [8]. The care-giving effect is the health effect on an individual providing, often burdensome, informal care to the patient. The family-effect is the health loss that is suffered by an individual due to the fact that someone in their social environment is ill, irrespective of whether they provide care to the patient [8]. Illness of a loved one results in “anxiety, worry, grief” which results directly in reduced health [8] page 292. These effects were investigated in a Dutch sample of 751 caregivers where the family effect was approximated to the general HRQoL of the patient (recorded on EQ-5D-VAS), and the care-giving effect was measured by the number of care-giving tasks [8]. The results showed that the two effects were distinguishable and individually associated with the HRQoL of caregivers [8].

The presence of the family effect is strengthened by evidence from a US sample which suggests that individuals who live with someone who is chronically-ill have a lower EQ-5D score than those who live alone or with people who are healthy [9]. To better understand the health spill-overs in the family effect on different family members an online survey was conducted on a US representative sample of 1267 adults and 102 adolescents who were living with a family member with a chronic condition [10]. They were asked to rate the spill-over effect of their family member’s HRQoL on their own HRQoL on a scale of 0-100 [10]. The results showed that the physical and emotional impact of illness on family members HRQoL varies by the nature of the relationship they have with the individual who has a chronic condition [10]. Having a parent with arthritis or depression is associated with a greater spill-over effect when compared to a spouse with the same conditions [10]. Similarly, having a child with cancer or depression is associated with a greater spill-over effect compared to having a spouse with either of these conditions. The age of the child may also affect the evaluation as a study by Prosser et al. (2005) found that adults indicated a greater willingness-to-pay for younger children compared to older children [11]. These results indicate that although considering the family effect in CUA is important, one needs to acknowledge that the size of the spill-over effect may vary and more work needs to be done to evaluate these effects [10].

In order to evaluate these spill-over effects further for economic evaluation the CarerQoL was developed in order to measure the care-related HRQoL in informal caregivers [6]. The instrument was modelled on the EQ-5D with seven dimensions measuring the burden of care and a Visual Analogue Scale (VAS) measuring happiness which was considered a valuation component [6]. The instrument was tested on a Dutch sample of 175 caregivers and was found to be feasible and displayed convergent and clinical validity [6]. Furthermore, the seven burden dimensions explained 37-43% of the variation in CarerQoL-VAS scores on multivariate analysis [6]. Instruments such as the CarerQoL have yielded important information in the spill-over effect of caring for an ill adult, but little work has been done in measuring the spill-over effect in caring for ill children. It has however been noted that providing care to a child with a chronic condition is burdensome and may affect the physical and psychosocial health of the caregiver negatively [12]. This is attributed to the fact that delivering this care is mostly unexpected, complex and often leads to extra expenses for medication and equipment as well as extended time for caring of the child [12].

In the few studies that have been conducted investigating the spill-over effect of caring for ill children it has been found that the caregiver’s HRQoL is affected by many factors, including the child’s perceived health vulnerability [13]. Caregivers of children with mental health problems have been shown to have a moderate level of depressive symptoms [14]. Furthermore, the mental HRQoL of caregivers of children with Autism Spectrum Disorder was lower than that of the general population, their physical HRQoL was however comparable [15]. Similarly, caregivers of children with asthma showed higher levels of anxiety when compared with the norm [16]. This may be attributed to the nature of the disease with frequent symptomatic episodes as well as the substantial caregiver responsibility in ensuring a clean and safe environment and regular medication use [16]. Accounting for these spill-over effects in health economic evaluations has proven challenging.

There are two theoretical frameworks which have been suggested to calculate this spill-over-effect in health care [7, 17]. Basu and Meltzer (2005), approach the calculation from the perspective of an individual who elects care based on maximizing their own utility in terms of survival and health status [17]. The suggested calculation: “total effect of an adverse health state = direct effect on patient’s utility +indirect effect on patient’s utility through family members’ utility +direct effect on family members’ utility” [17] page 759. The effect on a family members utility was measured using a modified time trade-off method (TTO) to estimate spill-over effects on the spouse of patient with prostate cancer [17]. The framework developed by Al-Janabi (2016), approaches the calculation from the perspective of a societal decision maker e.g. government agency, and maximizes health benefits for the population [7]. Al-Janabi’s framework (2016), incorporates health spill-over effects into the existing evaluation where the ratio of the total health from the patient and their family over the patient’s health only is subjected to two multiplier effect [7]. The one multiplier effect is defined by the value attained from providing a new health intervention t whereas the other is defined by the value attained from funding this new intervention [7]. CUA from a societal perspective may pose ethical considerations in resource allocations in that greater funding is allocated for people with dependents, informal caregivers or spouses at the expense of those without [7, 17].

Although Prosser et al. (2005) showed that the willingness-to-pay was higher for younger children there have been no other studies that evaluated the spill-over effect of very young children’s HRQoL on caregivers. We therefore wanted to explore the inter-relationship between the perceived HRQoL of the caregiver and that of the child The HRQOL of the caregiver was measured on the EQ-5D-3 L which consists of five domains of health, each with three levels of report, and a measure of general health on a VAS from 0 to 100. The EQ-5D-3 L has a preference based scoring system or an index score which is derived from the general population, the index score in this study is based on the United Kingdom TTO index score. The HRQoL of the child is rated by the caregiver on the TANDI which consist of six domains of health, each with three levels of report, and a measure of general health on a VAS from 0 to 100. We hypothesised that there would be a positive correlation between the VAS of the child and the caregiver and that this might be stronger in children who were acutely ill or still recovering from illness. This was shown in a study by Klassen et al. (2008), where caregivers of children with cancer experienced a greater negative impact on their HRQoL if their child’s treatment was more intense and if the child was more recently diagnosed with cancer [18]. We further expected that the correlation between the index score of the caregiver and the VAS of the child would be strongest in the caregivers of children with chronic illness as the long term physical care required might influence the functioning domains of the primary caregiver as shown in studies with caregivers of children with Cancer, Depression [10] Autism Spectrum Disorder [15], Cerebral Palsy [19], Spina Bifida [19], Congenital Abnormalities [20] and Asthma [16]. As it might be expected that the caregivers of children with acute or chronic illness may themselves be more likely to have chronic illness, most especially in relation to mental health, we needed to test whether this was so [15, 16]. Finally, we wanted to determine the predictive value of the caregiver’s general health score (measured on a VAS from 0 to 100) with respect to their reporting of their child’s general health score (measured on a VAS from 0 to 100), and vice versa. Thus what was the contribution of the VAS of the other member of the dyad to the variance in their respective VAS scores?

Thus, the aim of this study was to quantify the effect of the very young (1-36 months of age) child’s perceived general health as assessed by newly developed TANDI on the reporting of the caregiver’s own HRQoL as assessed by the EQ-5D-3 L. A further aim was to establish whether the health of the child, characterised by acutely-ill (AI), chronically-ill (CI) or general population (GP) was associated with the caregiver’s health.

## Methods

### Participants

The research settings included a tertiary level paediatric hospital, situated in Cape Town, South Africa, managing both AI and CI children. The hospital treats over 250,000 patients a year in both the acute and chronic services. Three open day care centres and three toddler play groups were included in the study. Caregivers of children aged between 1 month and 0 days and 35 months 30 days (1-36 months) accessing acute or chronic health care services or attending any of the participating day-care centres or toddlers groups were included. The caregiver of the child was defined as any person over the age of 18, who lived with the child and was wholly or partly responsible for the care of the child’s physical and emotional needs e.g. mother, father, aunt, uncle, grandparent, brother or sister.

Caregivers who were unable to read or write English were excluded as the TANDI (measuring the child’s HRQoL) was an English proto-type instrument and the validity and reliability was concurrently tested before translation into other languages. Caregivers of children who were medically unstable, terminally ill, or who were born prematurely and had not yet reached the corrected age of one month were excluded. An unstable child was classified as any child who was less than 24 h post admission to ICU, less than 24 h post-surgery or any child who had any acute changes in their medical condition.

In order to determine the minimum sample size of caregivers needed to determine the variance of the VAS score caused by dimension scores and the child’s perceived health the sample size calculation was based on regression analysis with an anticipated effect size of 0.15 using GPower version 3.1. The desired power level was set at 0.95; the number of predictors was set at the maximum number of 11 and the type 1 error was set at 0.05. The sample size of 179 was computed.

Caregivers of GP children were recruited from day-care centres and play groups. Research packs were sent to 112 caregivers inviting them to participate in the study. Caregivers of 67 children consented and returned the research packs. All of the caregivers of AI children who were approached and met the inclusion criteria consented to participate in the study. All 60 of the caregivers of AI children completed the study. All of the caregivers of CI children who were approached and met the inclusion criteria consented to participate in the study. All 60 of the caregivers of CI children completed the study. A total sample of 187 caregivers participated in the study.

### Measures

#### EQ-5D-3 L

The caregiver’s HRQoL was measured using the EQ-5D-3 L, an adult self-report measure assessing five dimensions of health: mobility (Mob), self-care (SC), usual activities (UA), pain/discomfort (P/D), anxiety/depression (A/D) and a rating of health status on a VAS [21, 22]. Each of the five dimensions of health have three levels of report: no problems, some problems and extreme or unable to do [21], [22]. The VAS is a vertical, graduated scale from worst imagined health state (0) to best imagined health state (100) on which the subject rates their overall health status [21], [22]. All ratings are made by the respondent based on their perceived health on the day of administration [21], [22]. The EQ-5D has been used and found to be valid in South Africa across health conditions as well as cultural and language groups [23–27]. South Africa does not locally derived preference based scores, thus the UK TTO preference based scores were used.

#### TANDI

The TANDI was developed as an experimental version modelled on the EQ-5D-Y proxy version 1 for children aged 1 month 0 days −36 months 0 days. The development of the instrument drew on results of a systematic review of the literature, cognitive interviews with caregivers of very young children, a Delphi study with experts in the field, development and finally the testing and validation of an alpha and beta draft of the instrument. This process resulted in a six item scale with three levels of report and a general rating of health on a VAS from 0 to 100. The descriptive dimensions include: movement, play, pain, relationships, communication and eating. The TANDI was found to be valid and reliable for use with children aged 1-36 months in South Africa. The content validity was established during the development of the instrument. Concurrent validity of the different items (dimensions) was tested between the TANDI and relevant items from the Ages and Stages Questionnaire (ASQ), FLACC (Faces, Legs, Activity, Cry, Consolability Observational Pain Scale) and NIPS pain scales (Neonatal Infant Pain Scale) and Diet History. The Kappa co-efficient ranged from 0.33 (fair) to 0.61 (moderate). The six items of the TANDI were tested for internal consistency and reliability and was reliable with Cronbach’s α = 0.83. Known groups were compared (construct validity) and the AI children had the lowest ranked VAS (median 60, range 0-100), indicating worst HRQoL and the GP group was significantly different from AI and CI (*p* < 0.01) but AI and CI were not different.

#### Contextual information

Contextual information was gathered on the relationship of the caregiver to the child, the health condition of the child and of the caregiver.

### Procedure

After ethical approval was obtained from the University of Cape Town Medical Research Ethics Committee and permission was granted from the children’s hospital and day-care centres the study commenced. Caregivers of children attending the day-care centres and play groups were sent a detailed description of the study before the study commenced. As pre-arranged with each of the day-care centres and play groups a research pack in an envelope was delivered for each child between the ages of 1-36 months. The research pack consisted of detailed information regarding the study, informed consent, a form capturing general information about the caregiver and child, the EQ-5D-3 L and the TANDI. The caregivers who consented to participate were requested to return the sealed envelope, with the completed research pack therein after a period of three days.

Caregivers of AI children were recruited individually from the in-patient wards of a children’s hospital. The recruitment process was done systematically throughout the hospital. Participants were first recruited the first floor medical ward from the first cubicle to the last cubicle in numerical order in each of the wards. The subsequent wards were done in the same manner from the first to the fourth floor of the hospital. The pattern was repeated until 80 caregivers had consented and participated in the study. The caregivers were given detailed information regarding the study and informed consent was taken, 24 h or later, post admission to the acute hospital. With the assistance of the researcher caregivers were asked to complete the research packs.

Caregivers of CI children were recruited from the waiting rooms of specialist clinics at the children’s hospital. These clinics included: neurology, cardiology, oncology, haematology, allergology, respiratory, rheumatology, developmental services and physiotherapy. Individual caregivers were approached from their position in the room; the caregiver closest to the left hand side of the door was recruited first and in a clockwise direction thereafter. After those caregivers were invited to participate, any new caregivers were approached in the order that they entered the waiting room. The caregivers were given detailed information regarding the study and informed consent was taken. With the assistance of the researcher, caregivers were asked to complete the research packs.

### Data management and analysis

The information from the contextual information, TANDI and EQ-5D-3 L was entered into an excel spread sheet under the code allocated to each individual.

Descriptive statistics were used to record the frequencies of responses to categorical data. As there were few responses in most dimensions at an extreme (three) level, the responses were dichotomised into “No Problems” and “Problems”. The results of the three groups (AI, CI and GP) were compared with regard to caregiver VAS, preference weight, child’s age and child’s VAS. Scatterplots and correlations were done to establish the relationship between the child’s VAS and VAS and preference based score of the caregiver. Finally forward step wise multiple regression analysis was done to establish the relative contribution of the VAS of the other member of the dyad to the VAS scores of the caregiver and the child. Forward stepwise regression models were developed with 1) the VAS score of the caregiver and 2) the VAS score of the child as dependent variables. The independent variables for the caregiver included dummy variables for the presence or absence of problems on the EQ-5D-3 L and the VAS score of the child. The independent variables for the child included dummy variables for each TANDI dimension and the VAS of the caregiver. As the age of the child was found to be significantly different across groups, this was included as an independent variable. Outliers whose residual scores were more than two SD from the mean were excluded. Analysis was performed using Statistica version 13.

## Results

### Caregiver EQ-5D-3 L results

The majority of caregivers across groups were mothers (90%) and there was no significant association between gender and groups (Chi-sq = 15.54 and *p* = 0.114). No data was collected regarding the age of the caregivers.

Medical conditions were reported by 44 caregivers and were equally distributed across caregivers of AI (23%), CI (23%) and GP (24%) children (*p* = 0.791). Most of the caregivers were healthy (76%) with a smaller number of them reporting illness (24%). There was no significant association between illness in the caregiver and illness in the child (AI, CI or GP) (Chi-sq = 0.007, *p* = 0.791). Within the ill group there were a high number of caregivers with HIV (23%), diabetes mellitus (16%), hypothyroidism (14%), hypertension (9%), generalised anxiety (9%) and depression (7%). Generalised anxiety was highest in caregivers of GP children and depression was equally distributed between the caregivers of AI, CI and GP children. The category of other included but was not limited to: tuberculosis, rheumatoid disease, haematological disease, lower back pain, migraines, hyperlipidaemia, endometriosis and pregnancy.

As seen in Table 1 the frequency of reporting problems in the dimensions was low across the caregivers, apart from the P/D and A/D dimensions, in which approximately 20% reported problems. As no respondent reported problems with SC, this dimension was excluded from analysis. Although caregivers of AI children reported the highest number of problems for dimensions of UA and A/D, there was no association found between the group and the percentage reporting problems in any dimension.

**Table 1:** Frequency of reporting problems in each dimension categorised by the classification of their child’s health

	AI Child	CI Child	GP Child	Total
	(*n* = 60)	(n = 60)	(*n* = 67)	(*n* = 187)
EQ-5D-3 L Problems with Mobility	2%	3%	6%	4%
EQ-5D-3 L Problems with UA	10%	5%	8%	7%
EQ-5D-3 L Problems with P/D	18%	18%	19%	19%
EQ-5D-3 L Problems with A/D	27%	17%	19%	21%

The mean preference based score (using the UK TTO values) was 0.89 (SD = 0.21) and ANOVA revealed no difference between the groups (F (2, 184) =1.47, *p* = .234). Similarly the mean VAS of the caregivers was 86.3 (SD = 15.5) and there was no difference between the groups (F (2, 184) =1.482, *p* = .223).

### Characteristics of the children

The mean age of the children was 18.7 months (SD = 10.6) and the ages differed significantly across groups (F (2, 184) =27.493, *p* < .001). The mean age across groups was AI 11.5 months (SD = 10), CI 20.5 months (SD = 10.7) and GP 23.5 (SD = 7.3), Post-hoc testing indicated that the AI children were significantly younger than the other two groups.

Most of the GP children did not have any medical diagnosis, but 16% of these children were reported as having a medical diagnosis including: asthma, allergies, breathe holding spells, cerebral palsy (hemiplegia), diarrheal disease, pneumonia and a throat infection. The majority of AI children had undergone general surgery and a number of children had multiple health conditions but the highest number of primary reasons for hospitalisation included: pneumonia, congenital heart disease, upper airway obstruction, neurosurgery intervention and diarrheal disease. The aetiologies for the CI children were also complex but include: cerebral palsy, developmental delay, epilepsy, haematology, oncology, and children with an upper airway obstruction requiring a tracheostomy.

The caregivers reported a higher number of AI children with problems in the play, pain and eating dimensions and more CI children with problems in movement, relationships and communication (Table 2). In every dimension, the GP children had the least number of children with reported problems and problems were associated with group in every dimension except eating. The dimension of eating had a high proportion of problems (25%) in the GP group. Thus a problem with eating was a more frequently reported problem across all three groups of children.

**Table 2:** Frequency of reporting problems for children in each dimension categorised by their health classification

	AI	CI	GP	Total	Chi-sq
	(*n* = 60)	(*n* = 60)	(*n* = 67)	(*n* = 187)	
TANDI Problems with Movement	40%	48%	5%	30%	***33.3 (p < 0.001)***
TANDI Problems with Play	42%	40%	5%	28%	***28.4 (p < 0.001)***
TANDI Problems with Pain	27%	11%	10%	16%	***7.4 (p = 0.024)***
TANDI Problems with Relationship	30%	32%	12%	24%	***8.4 (p = 0.015)***
TANDI Problems with Communication	37%	45%	12%	31%	***17.9 (p < 0.001)***
TANDI Problems with Eating	40%	30%	25%	32%	3.3 (*p* = 0.199)

Significant *p* values are bolded

**Fig. 1:**
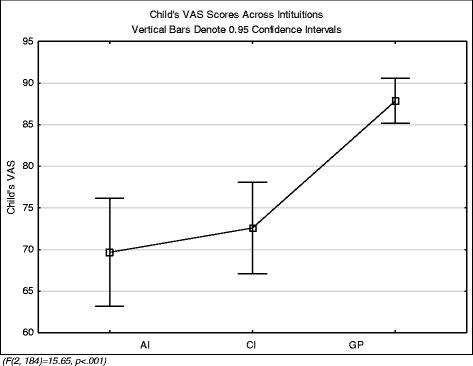
VAS comparison across groups

The mean VAS of the children was 77.1 (SD = 21.3) and this was significantly different across groups (F (2, 184) =15.65, *p* < .001)) (Fig. 1). Post-hoc analysis indicated that the GP children had a significantly higher VAS but that there was no significant difference between the other two groups.

### Relationship between the caregiver and child results

Categorised scatterplots were produced to depict the correlations between the VAS of the child and the VAS and preference based score value of the caregiver.

**Fig. 2:**
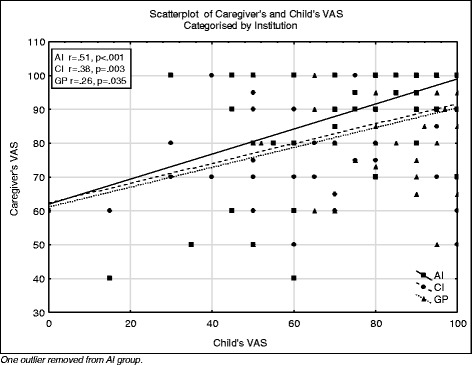
Scatterplot of caregiver’s and child’s VAS

There was a positive, significant correlation between the two VAS scores but it was strongest in the AI (*p* < 0.001) and weakest in the GP group (*p* = 0.035) (Fig. 2). It is also apparent that there were few GP caregivers or children whose VAS fell below 60.

**Fig. 3:**
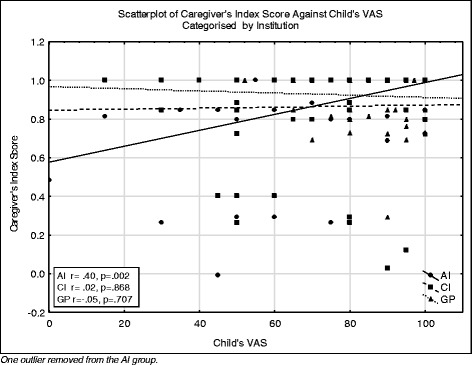
Scatterplot of the index scores of the caregiver and the VAS of the child

With regard to the preference based scores, only the AI correlation was significantly correlated (*r* = 0.40, *p* = 0.002) (Fig. 3). It can also be seen that apart from one GP caregiver, all the index scores below 0.6 were reported by the caregivers of the AI and CI groups.

Forward stepwise regression with the caregiver’s VAS score as dependent variable was computed. Residual analysis was performed to identify the participants whose scores were >2 standard deviations between predicted and observed. Seven outliers were excluded resulting in a model with an Adjusted R^2^ = 0.374 (Table 3).

**Table 3:** Regression analysis of the caregiver’s EQ-5D-3 L VAS score

	b*	Std.Err. of b*	b	Std.Err. of b	t(175)	*p*-value
Intercept			69.0	3.643	18.94	0.000
EQ-5D-3 L problems with A/D	−0.320	0.061	−11.8	2.249	−5.24	***< 0.001***
TANDI VAS	0.432	0.065	0.3	0.046	6.70	***< 0.001***
EQ-5D-3 L problems with P/D	−0.194	0.062	−7.3	2.339	−3.12	***0.002***
No Diagnosis in the Caregiver	−0.207	0.063	−6.6	2.002	−3.27	***0.001***
EQ-5D-3 L problems with Mob	−0.128	0.062	−9.8	4.723	−2.07	***0.040***

Significant *p* values are bolded

b* denotes standardized Beta regression coefficients

b denotes non-standardized Beta regression coefficients

*N* = 181 Adjusted R^2^ = 0.374

A summary of forward stepwise regression analysis indicates that the A/D dimension accounted for most variance (18%) and this was followed by the child’s VAS (11%), more than the presence of P/D, of a health condition and or mobility problems.

Additional models tested included the age of the child and admission to the AI group but these did not yield a better fit. A model was tested in which the child’s VAS was excluded and the AI variable was included but it resulted in a poorer Adjusted R^2^ (0.226) and the AI variable remained a non-significant predictor.

A similar analysis was performed to determine the influence of the caregiver’s VAS on his/her reporting of the child’s VAS. The dependent variable was the child’s reported VAS and the independent variables were the VAS of the caregiver, the age of the child and the presence of problems in each of the six dimensions of the TANDI. After the removal of 10 outliers, a model was developed which accounted for 54% of the variance (Adjusted R^2^ = 0.54) (Table 4).

**Table 4:** Regression analysis of the child’s TANDI VAS score

	b*	Std.Err. of b*	b	Std.Err. of b	t(168)	p-value
Intercept			51.5	6.458	7.97	0.000
TANDI Problems with Play	−0.128	0.081	−5.6	3.502	−1.59	0.115
EQ-5D-3 L VAS	0.324	0.054	0.4	0.067	6.03	***< 0.001***
TANDI Problems with Eating	−0.207	0.058	−8.5	2.411	−3.54	***0.001***
TANDI Problems with Movement	−0.255	0.073	−10.7	3.060	−3.50	***0.001***
TANDI Problems with Pain	−0.143	0.057	−7.4	2.956	−2.51	***0.013***
TANDI Problems with Relationships	−0.088	0.068	−4.0	3.109	−1.29	0.199
Age of Child in Months	0.072	0.053	0.1	0.096	1.36	0.175
TANDI Problems with Communication	−0.081	0.074	−3.4	3.079	−1.09	0.276

Significant *p* values are bolded

b* denotes standardized Beta regression coefficients

b denotes non-standardized Beta regression coefficients

*N* = 177 Adjusted R^2^ = 0.54

A summary of forward stepwise regression analysis indicated that play accounted for 25% of the variance and the caregiver’s VAS accounted for an additional 14% of the variance.

## Discussion

The results indicate that, as hypothesised, the perceived health states of the caregiver and the child are interrelated, and that an increase in ten points in the VAS score of the one would lead to an increase in three to four points in the VAS of the other. The correlation between the two sets of scores is most evident between the caregivers and children who are acutely-ill, particularly between the VAS of the child and the preference based score of the caregiver.

The sample of caregivers was generally representative of the South African population. According to Statistics South Africa 42.5% of children under five years of age live with their biological mothers only [28], thus it was not surprising that the majority of caregivers in the study where mothers. The occurrence of medical conditions was reported at 23% for all caregivers which is similar to the World Health Organisation (WHO) estimate of burden of disease in South Africa where 28% of the total burden is accounted for by non-communicable disease [29]. Furthermore, the conditions suffered by caregivers are in keeping with the major non-communicable diseases in South Africa which include cardiovascular disease, diabetes and mental illness [30]. The sample included a wide range of health states in children as well as caregivers with problems.

In addition, the responses of the caregivers, were typical of population based studies and broadly similar, e.g., to those found in population surveys in both China and Sweden in that the greatest number of problems were reported in the A/D and P/D dimensions and few in the SC dimension [31, 32]. Furthermore, the mean VAS scores were similar across countries: South Africa (86.3), Sweden (85) and China (79.4). Although South Africa does not have a preference based score set, the caregiver’s preference based scores were calculated using the UK TTO values and similar results were found between the South African sample with a mean index score of 0.89 compared to the Swedish sample with a mean index score of 0.84 [32].

One of the objectives of the study was to establish if children who were ill had caregivers who had health conditions and poorer HRQoL. In fact, there was no association between the presence of a health condition and group and the VAS and preference based scores of the caregivers were not different between the three groups. Furthermore, the reporting of mental health conditions was in fact higher in caregivers of GP children than CI children. This was not expected as literature has noted the increased burden of care for a chronically-ill individual [9, 10, 12–16]. This has been shown in children diagnosed with asthma [12, 16], genetic conditions [13], Autism Spectrum Disorders [15], Spina Bifida [19] and mental health concerns [14]. It was thus surprising that care for CI children in this sample did not result in either a decreased VAS or index score in the caregiver. This could be due to hedonistic adaptation [33] or a response shift from the caregiver’s perspective [34]. Response shift has been found in self-report from children who are chronically ill where it is thought that the children’s internal standards and conceptualisations change due to their long standing condition [35]. Caregivers of chronically ill children may experience a similar response shift to that of their children resulting in a changed perception of their HRQoL and thus scores similar to caregivers of GP children. On the other hand, as the chronically ill children were not institutionalised and were mostly ambulant, the burden of care in this sample may have been less than in other studies.

With regard to the measurement of the HRQoL of the children, the TANDI performed well. The VAS did discriminate between GP and the ill children and the dimensions indicated more problems in the AI and CI groups. Based on the nature of the AI and CI, the frequencies of problems seemed intuitively correct, with more problems in movement and communication reported in the CI Group and more problems with pain and eating reported in the AI Group. In addition, multiple regression analysis indicated that the coefficients of all six dimensions were negative and reduced the VAS score, albeit not significantly in every case. The three dimensions that decreased the score most were Eating, Movement and Pain, all of which were more evident in the AI and CI children.

Although there was no difference in VAS or preference based score between the caregivers of the three groups, there was a significant correlation found between the VAS scores of both members of the dyad. In contrast, the correlation between the VAS score of the child and the preference based score of the caregiver was only significant in the AI groups. This is likely to be a reflection of the spread of scores and the greater variance in the AI children. As the children were perceived to improve, it appears that the preference based score of the caregiver improved. Consideration should be given to whether an artificial correlation between caregiver’s and child’s health status was introduced with the use of caregivers as proxy raters of their child’s health [9, 19]. This might have been the case, but does not explain why the correlation was higher in the AI group.

The spill-over effect was also demonstrated by the regression analysis. It was thought that the relationship between the two VAS scores would most likely be mediated by the A/D dimension in the caregivers [15, 16] and problems in this dimension did indeed reduce the VAS considerably (12%). However, over and above the A/D, the effect of the child’s VAS had an effect which accounted for a considerable amount of the variance, with a change of 10 points resulting in a reduction of 3-4% in the VAS score of the other member of the dyad. There is clearly a spill-over effect but it is not as large as expected.

Hoefman 2013, suggest that if the health effects in the carer are measured on the same instrument as that of the patient the QALYs for the two individuals can simply be aggregated for CUA [5] and this may be a useful line of inquiry. Careful consideration would however need to be given to the generic HRQOL measure used as well as its sensitivity in detecting caregiver concerns [5]. Tilford and Payakachat (2015), examined direct elicitation techniques to measure family spill-over effects [17, 36]. They conducted a study of caregiver spill-over effect associated with sleep treatment for children with Autism Spectrum Disorder [36]. Caregiver results on both the SF-6D and the EQ-5D showed similar marginal effects showing that caregiver HRQoL would improve with treatment of the child as well as improvement of the child’s health [36]. Similarly a study using generic instruments in measuring preference based scores in both children with spina bifida (measured on HUI: 2 proxy) and their caregivers (measured on the Quality of Well-being scale) showed that less disability in children is associated with clinically significant higher preference-weighted health states in the caregiver [19]. It is suggested that using the same generic instrument in both groups may strengthen this relationship for CUA. However this is clearly impossible when the HRQoL adults and infants or toddlers are compared.

The study may have been limited by the fact that the perceived rating of the child’s VAS by the caregiver could have been impacted by the caregivers own HRQoL. The proxy rating could further have been clouded by the caregiver’s expectations of the child, their definition of HRQoL and their understanding of the child’s illness and its sequelae [37]. An additional limitation included selection or completion biases as some GP caregivers may have been more motivated to return the research pack versus those that were recruited but did not return the pack. The reason for problems experienced per domain in the caregiver was not sought; these reasons could have helped understand whether problems were associated with their child. It is thus recommended that future studies investigate the difference in effect if the caregiver proxy or an unrelated adult proxy rates their child’s HRQoL. As well as examine the reasoning behind the caregivers reporting problems on dimensions of the EQ-5D-3 L to clarify whether the problems reported are in fact due to the child’s health.

## Conclusion

There is clearly a relationship between the caregiver’s perceived HRQoL and that of the infant and young child and this is most apparent in AI children. Of interest to CUA is that the preference based score of the caregiver was also influenced by his/her perception of the child’s general health. This leads us to question how to factor in this spill-over effect. Should a compound instrument be developed for this age group? Are the gains in HRQoL in child and caregiver somehow additive and, once there are preference based scores for the infants and young children the gains should simply be summed? Or should the valuation of infant health states be done, using health states that reflect both caregiver and child’s state. It is recommended that future research investigates this effect with regards to CUA calculations.

## Abbreviations

A/D: Anxiety/depression
AI: Acutely-Ill
ASQ: Ages and Stages Questionnaire
CI: Chronically-Ill
CUA: Cost Utility Analysis
FLACC: Faces, Legs, Activity, Cry, Consolability Observational Pain Scale
GP: General population
HRQoL: Health Related Quality of Life
Mob: Mobility
NIPS: Neonatal Infant Pain Scale
PD: Pain/discomfort
QALYs: Quality Adjusted Life Years
SC: Self-care
TTO: Time trade-off
UA: Usual activities
UK: United Kingdom
VAS: Visual Analogue Scale
WHO: World Health Organisation
